# Autophagy Triggered by Oxidative Stress Appears to Be Mediated by the AKT/mTOR Signaling Pathway in the Liver of Sleep-Deprived Rats

**DOI:** 10.1155/2020/6181630

**Published:** 2020-02-13

**Authors:** Yongmei Li, Yuan Zhang, Guang Ji, Yiwei Shen, Nan Zhao, Yuhan Liang, Zihan Wang, Mengqi Liu, Laixiang Lin

**Affiliations:** ^1^NHC Key Laboratory of Hormones and Development (Tianjin Medical University), Tianjin Key Laboratory of Metabolic Diseases, Tianjin Medical University Chu Hsien-I Memorial Hospital & Tianjin Institute of Endocrinology, Tianjin, China; ^2^Basic Medical College, Tianjin Medical University, Tianjin, China; ^3^Qinghai Institute for Endemic Disease Prevention and Control, Qinghai, China

## Abstract

Sleep deprivation adversely affects the digestive system. Multiple studies have suggested sleep deprivation and oxidative stress are closely related. Autophagy can be triggered by oxidative stress as a self-defense strategy to promote survival. In this study, we investigated the effects of sleep deprivation on liver functions, oxidative stress, and concomitant hepatocyte autophagy, as well as the associated pathways. Enzymatic and nonenzymatic biochemical markers in the serum were used to assess hepatic function and damage. To evaluate the occurrence of autophagy, expression of autophagy-related proteins was tested and autophagosomes were labeled. Additionally, methane dicarboxylic aldehyde (MDA), antioxidant enzymes, and the protein kinase B (AKT)/mammalian target of rapamycin (mTOR) signaling pathway were analyzed using chemical methods and a Western blot. Serum alanine transaminase, aspartate aminotransferase, and alkaline phosphatase increased in sleep-deprived rats. Total protein and albumin abundance was also abnormal. Sleep deprivation induced histopathological changes in the liver. The superoxide dismutase level decreased significantly in the liver of sleep-deprived rats. In contrast, the MDA content increased in the sleep deprivation group. Moreover, the microtubule-associated protein 1 light chain 3 beta (LC3B) II/I ratio and Beclin I content increased considerably in the sleep-deprived rats, while p62 levels decreased. Sleep deprivation apparently inhibited the AKT/mTOR signaling pathway. We conclude that sleep deprivation can induce oxidative stress and ultimately cause liver injury. Autophagy triggered by oxidative stress appears to be mediated by the AKT/mTOR pathway and plays a role in relieving oxidative stress caused by sleep deprivation.

## 1. Introduction

Sleep deprivation (SD) refers to the inability to achieve adequate undisturbed night sleeps because of environmental or personal reasons. In humans, SD is associated with several adverse effects, including impaired learning and memory, physiology, psychology, and immune functions [1, 2]. Sleep reportedly has an antioxidative function [3], and previous studies revealed that SD alters systemic and brain energy metabolism [4, 5], possibly because of an accumulation of reactive oxygen species (ROS). Increased oxidative stress is one of the most important biological consequences of SD, ultimately leading to a series of negative effects, such as abnormal cognition and immunity, and diseases in the nervous, cardiovascular, and gastrointestinal systems [6–8]. Oxidative stress results from the inability to eliminate excess ROS, which is produced during normal cellular metabolism, because of a relative deficiency of enzymatic and nonenzymatic antioxidants [9, 10]. This imbalance may damage important biomolecules and organs or even the entire organism. Multiple studies have confirmed the close relationship between SD and oxidative stress [11, 12]. Valvassori et al. [13] proved that paradoxical sleep deprivation (PSD) induces hyperactivity (i.e., mania-like behavior) in mice by increasing lipid peroxidation and oxidative damage to DNA, while also disrupting antioxidant enzymes in the frontal cortex, hippocampus, and serum.

Autophagy is a mechanism that protects cells from injury via the degradation of dysfunctional organelles and misfolded or aggregated proteins. Additionally, it functions as a self-defense strategy that promotes cell survival by preventing apoptosis, necrosis, and pyroptosis [14, 15]. Moreover, autophagy can be triggered by oxidative stress. As the product of oxidative stress, ROS at low levels can serve as a signaling molecule that oxidizes the components of diverse pathways that lead to growth and survival. Furthermore, ROS functions as a signaling molecule in what is essentially a survival pathway that results in the formation of autophagosomes [8, 16].

Studies have indicated that autophagy can be activated by several pathways [17, 18]. Protein kinase B (AKT) is an important regulator of survival signals responsive to multiple stimuli inside and outside of cells. The associated mammalian target of rapamycin complex 1 (mTORC1) is a unique molecular transducer of cellular needs, which can recognize both glucose and amino acid signals. Additionally, AKT can phosphorylate related substrates that activate mTORC1. The resulting active mTORC1 can control the activity of eukaryotic initiation factors and eukaryotic elongation factors by phosphorylating p70 S6 kinase (p70S6K). This series of reactions may be considered as an AKT-mTOR-p70S6K signaling pathway, which inhibits autophagy [19, 20]. An oxidative signal is partially dependent on phosphatidylinositol 3-kinase (PI3K) and helps to inhibit the AKT-mTOR-p70S6K signaling pathway [21].

The liver exhibits a powerful compensatory ability and is highly resistant to oxidative stress. Furthermore, various antioxidant enzymes are highly abundant in the liver. There are relatively few reports describing liver damage induced by SD [6]. A few studies have shown that, in response to SD, serum alanine transaminase (ALT), aspartate aminotransferase (AST), and total bilirubin contents increase and liver cytokines are altered; these changes are indicative of liver damage [6]. In this study, the effects of SD on liver functions, oxidative stress, and concomitant hepatocyte autophagy in rats were investigated.

## 2. Materials and Methods

### 2.1. Animals and Diet

Forty healthy adult male Wistar rats (9-week-old, 300–350 g) were purchased from the animal center of the Military Medical Sciences Academy of the People's Liberation Army (Permission No. SCXK-(A) 2012-0004), after which they were housed in a standard laboratory room set at 23 ± 1°C and 55 ± 5% humidity, with a 12 h light/12 h dark cycle (lights on at 8:00 am). The rats were provided rodent chow (GB 14924.3-2010) and water *ad libitum*. Animal procedures were approved by the Institutional Animal Care and Use Committee of Tianjin Medical University, which is in accordance with the NIH Guide.

### 2.2. Reagents

Serum total protein (TP), albumin (ALB), ALT, AST, and alkaline phosphatase (ALP) test reagents were purchased from Roche (Shanghai, China). Methane dicarboxylic aldehyde (MDA, Catalog no.A003-1-2), glutathione peroxidase (GPx, Catalog no. A005-1-2), and superoxide dismutase (SOD, Catalog no.A001-1-1) test kits were purchased from the Nanjing Jiancheng Bioengineering Institute (Nanjing, China). Primary antibodies for the following antigens were used: *β*-actin (ABclonal Technology, Wuhan, China), microtubule-associated protein 1 light chain 3 beta (LC3B; Sigma-Aldrich, St. Louis, MO), p62 (Abcam, Cambridge, MA), LC3A, Beclin I (Proteintech Technology, Wuhan, China), mTOR, phospho-mTOR^(Ser2448)^, AKT, phospho-AKT^(Thr172)^, p70 S6 kinase, and phospho-p70 S6 kinase(Thr389) (Cell Signaling Technology, Danvers, MA), and a DAB (Catalog no. AR1000) kit was purchased from Boster Biological Technology Co., Ltd. (Wuhan, China). RIPA buffer was purchased from Solarbio Biological Technology Co., Ltd. (Beijing, China) and the PhosSTOP phosphatase inhibitor cocktail from Roche (Mannheim, Germany). Protein assay kit (Biomed, Beijing, China, Catalog no. PA101-01), secondary antibody (horseradish peroxidase- (HRP-) conjugated anti-rabbit/mouse IgG; Proteintech Technology, Wuhan, China), polyvinylidene difluoride membranes (0.22 *μ*m pore size; Millipore, Burlington, MA), and Immobilon Western Chemiluminescent HRP Substrate (Millipore, Burlington, MA) were used in Western blotting.

### 2.3. Experimental Design

#### 2.3.1. SD Procedure

**Figure 1:**
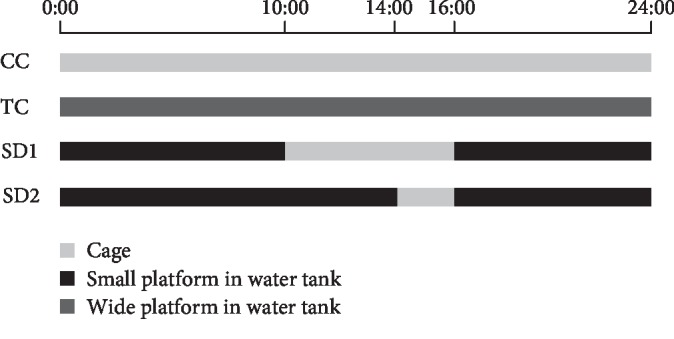
Timeline of SD procedure.

Rats were acclimated for 1 week before starting the experiments. They were then randomly allocated into the following four groups (10 rats in each group): cage control (CC) group, wide platform control (TC) group, sleep deprivation group 1 (SD1), and sleep deprivation group 2 (SD2). The rats in the SD groups underwent sleep deprivation according to a slightly modified version of the “platform on the water” method [22], but the rats in the CC group did not. A small circular platform (diameter, 63 mm; height, 80 mm) was positioned at the center of a transparent water tank (30 × 30 × 30 cm), which was filled with water (to 2 cm below the platform level). The rats were placed on separate platforms for SD. Specifically, the rats would awaken after falling into the water because of muscle tone loss during sleep. The animals in the SD1 and SD2 groups were deprived of sleep for 18 h (4:00 pm to 10:00 am) and 22 h (4:00 pm to 2:00 pm), respectively, after which they were allowed to sleep for 6 h and 2 h per day, respectively. To control the nonspecific effects of environmental factors, the rats in the TC group were placed in an identical tank equipped with a wide platform (diameter, 160 mm) that enabled them to move and sleep freely and were subjected to a sham intervention in a similar water environment. The animals in the TC group were otherwise intervened the same as the animals in the CC group (Figure 1). This experiment lasted for 21 days. Dietary intake was monitored on the day before the end of the experiment.

#### 2.3.2. Biological Sample Collection

At the end of the experiment, the blood was taken from the femoral artery of rats under anesthesia. The liver was excised from the abdominal cavity and weighed, then the left lobe of the liver was selected for histological and biochemical detection.

#### 2.3.3. Serum Biochemical Analysis

Serum samples were collected by centrifugation (3000 × g, 10 min) and then stored at −80°C. The AST, ALT, and ALP contents were analyzed according to the appropriate kit protocol.

#### 2.3.4. Detection of Oxidative Stress and Antioxidant Enzymes

The MDA content and the SOD and GPx levels (i.e., antioxidant enzymes) in the liver tissue were detected according to the appropriate kit protocol.

#### 2.3.5. Histological Assessment of the Liver

Liver tissue was excised, fixed in 10% phosphate-buffered saline-buffered formalin, and embedded in paraffin. The paraffin-embedded liver sections (4 *μ*m thick) were stained with hematoxylin-eosin. Histological changes were observed with a microscope (Olympus, Tokyo, Japan).

#### 2.3.6. Western Blotting

The liver tissues were homogenized in a RIPA buffer containing PhosSTOP phosphatase inhibitor cocktail. The homogenized tissue was incubated on ice for 15 min and centrifuged at 13,000 × g for 10 min at 4°C. The supernatant was collected and the pellet was discarded. The protein content in the tissue extract was measured using a bicinchoninic acid protein assay kit. An aliquot of the protein solution (corresponding to 50 *μ*g) was mixed with a loading buffer, heated at 100°C for 10 min, and then separated by sodium dodecyl sulfate-polyacrylamide gel electrophoresis (8–15%). The separated proteins were transferred to polyvinylidene difluoride membranes, which were then incubated in a blocking solution (5% nonfat milk in Tris-buffered saline containing 0.1% Tween-20 (TBST), pH 7.4) for 1 h at room temperature. Primary antibody was added to the membranes, which were incubated overnight at 4°C. The membranes were washed three times with TBST and subsequently incubated with secondary antibody for 2 h at room temperature. The membranes were washed three times with TBST, after which the protein bands were visualized with an Immobilon Western Chemiluminescent HRP Substrate. Protein abundance was normalized based on the amount of *β*-actin or the phosphorylated protein/total protein ratio.

#### 2.3.7. Immunohistochemistry Test

The IHC test procedure followed in detail the instructions of the kit. Paraffin sections were dewaxed routinely with water and incubated for 10 minutes with 3% H_2_O_2_ to deactivate the endogenous catalase. Then, the antigen was repaired by microwave. The sections were put into 0.01 mol/L citrate buffer solution (pH 6.0), heated for 10 minutes in a microwave oven, then cooled to room temperature and washed with 0.1 mol/L PBS for 3 times (5 minutes every time). Then, they were added in properly diluted antibody from rabbit, incubated overnight at 4°C, and washed with 0.1 mol/L PBS for 3 times (5 minutes every time). Sections was stained by DAB in a lucifugal condition at room temperature, washed with water sufficiently, redyed, dehydrated until hyaloid, mounted, and observed with a microscope.

#### 2.3.8. Statistical Analysis

All data are expressed as the mean ± standard deviation. Statistical analyses were performed using SPSS version 13.0 software (SPSS Inc., Chicago, IL). Data were compared according to a one-way analysis of variance followed by the least significant difference/Tamhane's multiple comparison test. A *P* value < 0.05 was adopted as the threshold for significance.

## 3. Results

### 3.1. Food Consumption and Changes in Hepatic Function in Sleep-Deprived Rats

**Figure 2:**
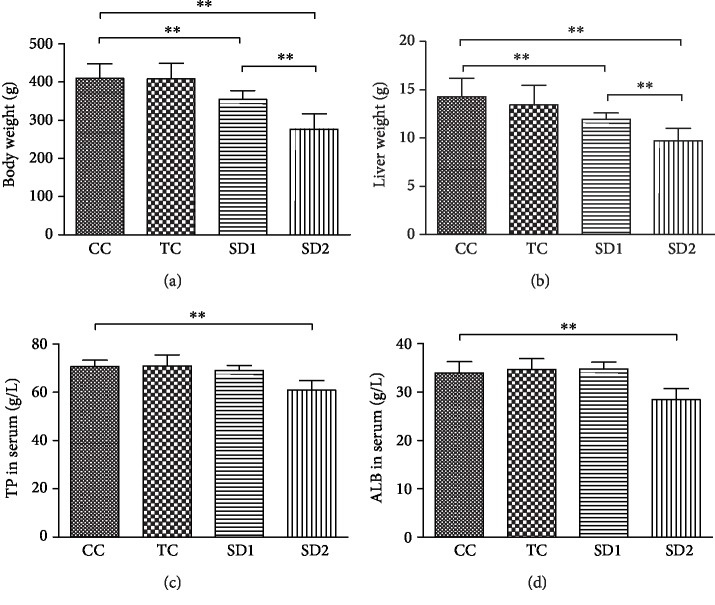
SD affected liver function. Both body (a) and liver weight (b) decreased in the SD groups. At the same time, TP (c) and ALB (d) in serum reduced significantly in the SD2 group (mean ± SD, *n* = 10 for each group). Above protein changes in serum revealed a weakened function. ^∗∗^Significantly different, *P* < 0.01.

**Figure 3:**
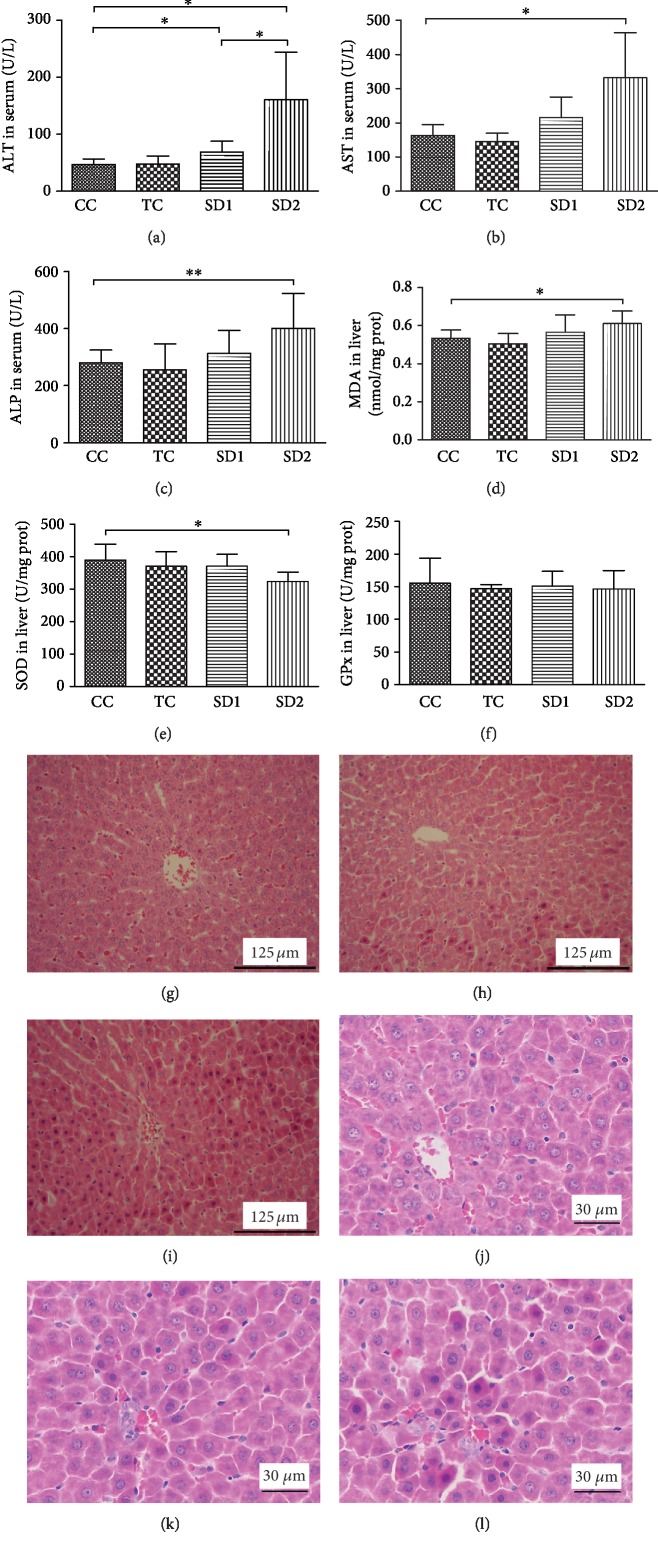
SD induced liver damage. Rats in four groups were challenged with SD or control. Serum ALT (a), AST (b), and ALP (c) levels were shown as mean ± SD (*n* = 10 for each group). MDA content (d), SOD activity (e), and GPx (f) activity in liver tissue were detected in the meantime (mean ± SD, *n* = 10 for each group) ^∗∗^*P* < 0.01 and ^∗^*P* < 0.05, based on the comparison with rats in the CC group. Histopathological changes were found in the rat liver on exposure to SD. Representative light microphotographs (magnitude 100x in (g–i) and 400x in (j–l)) of HE staining of liver: (g, j) control liver in the CC group; (h, k) liver in the SD1 group; (i. l) liver in the SD2 group.

Food consumption increased in the SD1 (21.60 ± 1.00 g/d) and SD2 (47.50 ± 4.93 g/d) groups, which was significantly more than that in the CC (19.58 ± 1.32 g/d) and TC (19.75 ± 1.50 g/d) groups (*allP* < 0.01). The TP and ALB contents (nonenzymatic biochemical parameters) in the SD2 group were abnormal compared with the corresponding contents in the CC group (Figure 2). The extent of the hepatic damage caused by SD was estimated by quantifying the biochemical parameters, including enzymatic and nonenzymatic markers. The observed increase in the hepatic enzyme contents (serum ALT, AST, and ALP) in the SD groups clearly reflected the SD-induced damage (Figures 3(a)–3(c)). Compared with the corresponding levels in the CC group, all of the changes to the test parameters were significant (*P* < 0.05 or *P* < 0.01) in the SD2 group, whereas only the change in ALT content was significant (*P* < 0.05) in the SD1 group.

### 3.2. Effect of SD on the Liver Antioxidant Status

Oxidative stress contributes to the mechanism underlying liver injury. Thus, we first examined the oxidative stress in the harvested rat livers by measuring MDA production and antioxidant enzyme levels. The hepatic antioxidant and lipid peroxidation levels were affected by SD, as indicated by the changes to the biochemical markers SOD, GPx, and MDA (Figures 3(d)–3(f)). The liver SOD level significantly decreased (*P* < 0.05) in the SD2 group. In contrast, the MDA content increased in the SD2 group. The liver GPx level showed no significant change in the SD groups.

### 3.3. Histopathological Analysis of Tissues

Macroscopic observations of liver sections suggested that the anatomical structures were normal in the rats of the CC and TC groups. However, histological analyses revealed some changes in the rats of the SD groups. In the SD1 group, the structures of the hepatic lobule and hepatic cell cords were normal, but the volume of some hepatocytes was smaller than that in the CC group. Additionally, the cytoplasm appeared to be affected by acidophilic degeneration, and the heterochromatin content in nuclei increased significantly. In the SD2 group, the histopathological changes were more obvious, with some hepatocytes exhibiting the early signs of hydrophobic change (Figures 3(g)–3(l)).

### 3.4. Enhanced Autophagy in the Liver of Sleep-Deprived Rats

**Figure 4:**
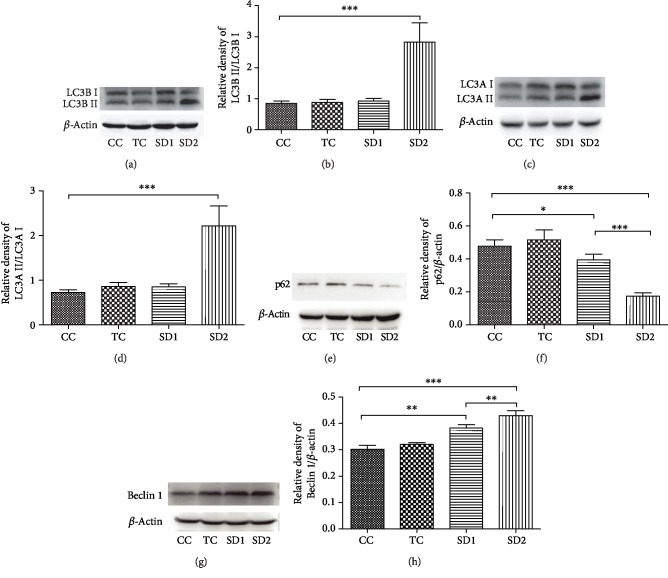
SD increased autophagy activity in liver. (a–d) The images presented were the expression of LC3. Bars represent the means of the LC3 II/LC3 I ratios. (e–h) Representative expression of protein levels of p62 and Beclin 1 were detected by Western blotting. The relative densities of the bands in each lane were analyzed and normalized to *β*-actin. The results were mean values ± SD (^∗^*P* < 0.05, ^∗∗^*P* < 0.01, and ^∗∗∗^*P* < 0.001).

**Figure 5:**
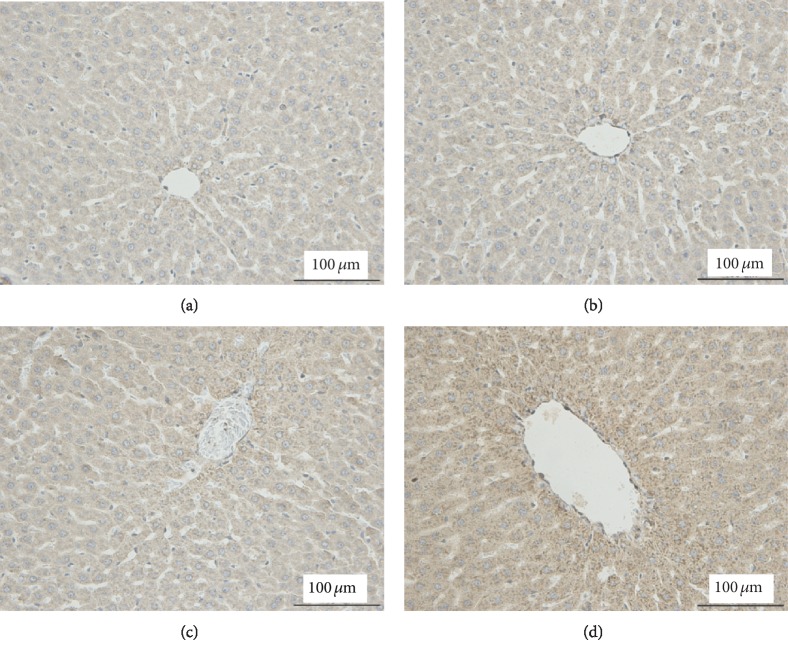
Autophagosome increased in SD liver. (a–d) The images presented were the autophagosome labeled with anti-LC3 antibodies by IHC. The autophagic vacuoles resided more in the SD-treated hepatocytes.

Autophagy is generally considered to be a protective mechanism in response to various stresses. To determine the autophagy status in the liver tissue of sleep-deprived rats, the abundance of the following autophagy-related proteins was analyzed by Western blotting: LC3A, LC3B, sequestosome (p62/SQSTM1), and Beclin I. The results indicated that the LC3B and LC3A II/I ratios were considerably higher in the SD2 group than in the CC group (Figures 4(a)–4(d)). Additionally, compared with the corresponding levels in the CC and TC groups, Beclin I and p62 contents increased and decreased, respectively, in the SD groups, especially in the SD2 group (Figures 4(e)–4(h)). Autophagosome labeled by anti-LC3 increased in SD liver (Figures 5(a)–5(d)). These findings indicated that SD enhanced autophagy in the liver.

### 3.5. The AKT/mTOR Signaling Pathway Contributes to SD-Induced Autophagy

**Figure 6:**
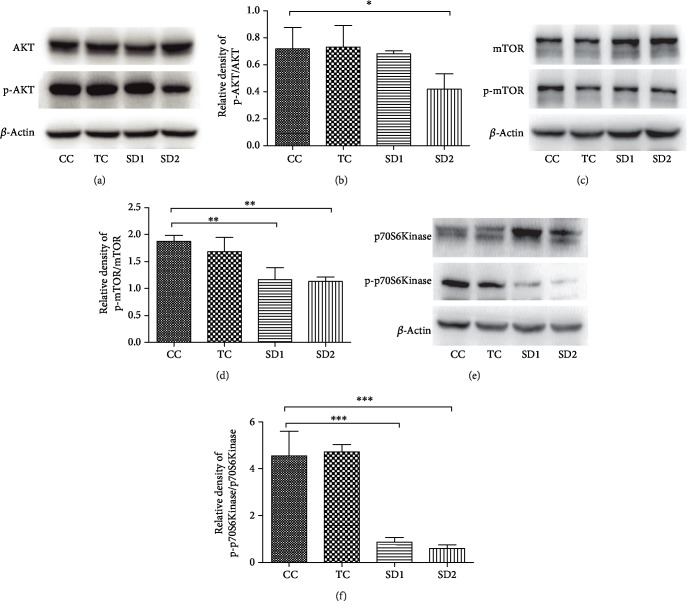
SD-induced autophagy was mediated by the AKT/mTOR pathway in the liver. The protein levels of AKT, mTOR, and p70S6Kinase were detected by Western blotting. The ratios of phospho-AKT vs. AKT, phospho-mTOR vs. mTOR, and phospho-p70S6Kinase vs. p70S6Kinase are shown. The results were mean values ± SD (^∗^*P* < 0.05, ^∗∗^*P* < 0.01, and ^∗∗∗^*P* < 0.001).

The AKT/mTOR signaling pathway is an important regulator of autophagy. To further investigate the possible molecular mechanism underlying SD-induced autophagy in the liver, Western blotting was used to analyze the AKT, mTOR, and p70S6K contents. The results suggested that SD can inhibit the AKT/mTOR signaling pathway. The phospho-AKT^(Thr172)^/AKT ratio was significantly lower in the SD2 group than in the other groups and appeared to be lower in the SD1 group than in the CC group, but the difference was not significant (Figures 6(a) and 6(b)). Additionally, mTOR is a major regulator of the autophagy pathway, and p70S6K is a key downstream target of mTOR. The mTOR and p70S6K signals decreased considerably in the SD groups (Figures 6(c)–6(f)). Moreover, the phospho-mTOR^(Ser2448)^/mTOR and phospho-p70S6K/p70S6K ratios also decreased in the SD groups. These findings suggested SD can inhibit the AKT/mTOR signaling pathway, which may contribute to increased autophagy in the liver.

## 4. Discussion

In this study, rats were deprived of sleep as previously described [23, 24]. Additionally, the TC group was used as a control for the confounding factors inherent to this method. Although there is some controversy in the use of this control [25], it may be appropriate for most of the confounding factors. Our results suggest that the confounding factors did not adversely affect the reliability of our results.

SD can damage the liver, and many enzymatic and nonenzymatic biochemical markers can be used to assess hepatic damage. Following structural damage to hepatocytes, the enzymatic markers ALT and AST enter the bloodstream [26]. Therefore, serum ALT activity is the most frequently used biomarker for detecting hepatic damage (i.e., clinical chemistry gold standard) [27]. However, AST is also used as a biomarker to assess liver function. In the current study, we detected elevated serum ALT and AST levels in the SD groups. Alkaline phosphatase, which is an extracellular hydrolytic enzyme, is a cholestatic induction marker of hepatobiliary origin that is eliminated in the bile [28]. Additionally, ALP content is used as a liver function biomarker. The elevated ALP levels in the SD groups were indicative of hepatobiliary dysfunction.

Protein synthesis is one of the most essential functions of a healthy liver. Thus, the TP level can be used to distinguish between normal and damaged livers [29]. Furthermore, ALB is a major protein in circulation, and a low serum ALB level is an indicator of hepatotoxicity. Hypoproteinemia prefigures impaired protein synthesis [30]. Our data indicated that the TP and ALB contents decreased in response to the SD treatment, which reflected the decline in the protein synthesis function of the liver [31]. Additionally, the observed histological alterations were consistent with the changes described above.

Oxidative stress has been implicated in the pathophysiology of many system function disorders. However, natural or synthetic antioxidant molecules may prevent these conditions and promote human health [32]. Antioxidant enzymes scavenge free radicals to maintain cell stability [33]. The oxidative stress status due to PSD has been studied in the whole brain for decades. Decreased GSH levels have been detected in specific brain regions (i.e., hypothalamus and thalamus), implying these regions may be susceptible to oxidative stress during PSD [34]. A recent study confirmed that SD predisposes the liver to the adverse effects of oxidative stress and phospholipid damage [35].

Oxidative stress in hepatic cells triggers a self-protection mechanism, leading to the elevated activity of hepatic antioxidant enzymes, such as SOD and GPx, which are ROS scavengers produced by cells to repair damage due to ROS [36]. Specifically, SOD catalyzes the dismutation of the superoxide anion to hydrogen peroxide and water [37]. A decrease in SOD levels may be the result of an inactivation of the enzyme or an increase in superoxide radical levels resulting from lipid peroxidation. The hepatic lipid peroxidation level is affected by SD, as indicated by the changes to biochemical markers such as MDA [38]. As the final product of lipid peroxidation, increases in MDA content lead to hepatic damage. In this study, the MDA level was notably higher in the SD2 group than in the CC group. These results imply that SD may promote the formation of oxidants and induce oxidative changes to lipids, ultimately resulting in altered membrane function, protein damage, and inhibited intracellular antioxidant defenses [39]. These observations are consistent with the earlier described changes to biochemical parameters.

Autophagy is a dynamic cellular process essential for the renewal and function of cells, and it reflects the state of an organ [40]. The liver is rich in lysosomes and undergoes substantial autophagy, which is precisely regulated by hepatic metabolic pathways. As indicated herein, the ROS level increases in sleep-deprived rats. Previous investigations have confirmed that autophagy can be triggered by oxidative stress to enhance cell survival [41, 42].

If autophagy cannot meet the demand for the clearance of old and damaged subcellular structures, high ROS levels can damage proteins, membrane lipids, nuclear DNA, and other cell molecules [43, 44]. However, few studies have assessed the effect of SD on autophagy in the liver. Thus, we examined the effects of SD on liver autophagy. The LC3 II protein is the lipid-conjugated form of LC3 located on the autophagosome membrane, and increasing LC3 II levels appear to correspond with increasing autophagy activity. In this study, we observed that the LC3 II content obviously increased in the SD2 group, suggesting there was an increase in the conversion of LC3 I to LC3 II, which is required for the formation of autophagosomes. To confirm this, we analyzed the p62 and Beclin I contents. A previous study revealed that p62, which is a selective autophagy substrate, tags protein aggregates via its ubiquitin-associated domain and recognizes and ubiquitinates the autophagy protein LC3 II [45]. Beclin I initiates autophagy and is used as a marker for monitoring the onset of autophagy. We observed that the p62 and Beclin I contents decreased and increased, respectively, in the SD groups. These findings suggest that SD may enhance autophagy in the liver.

The mTOR signaling pathway plays a significant role in inhibiting autophagy initiation and regulating autophagosome maturation. This pathway can integrate some autophagic signals, including starvation, growth factor deprivation, and oxidative stress [46]. Additionally, mTOR is an important negative regulator of autophagy that controls the expression of autophagy-associated genes [46]. Moreover, p70S6K, which is a major downstream target of the mTOR pathway, is essential for regulating cell proliferation and survival [47]. The AKT/mTOR pathway may contribute to the autophagy-mediated protection against oxidation. Consequently, we speculated that AKT/mTOR signaling might be involved in the autophagy induced by SD. In the present study, we investigated the main proteins of the AKT/mTOR pathway. The abundance of the core components of this pathway in the SD groups differed from that in the other groups. Furthermore, the phosphorylated AKT, mTOR, and p70S6K levels clearly decreased in the SD groups. Therefore, our results combined with previously reported data suggest that increased autophagy in response to SD may be mediated via the inhibition of the AKT/mTOR signaling pathway.

## 5. Conclusion

In summary, the data presented herein imply oxidative stress, autophagy, and liver damage are related. On the basis of our data, we speculate that SD can induce oxidative stress and damage the liver. Moreover, autophagy accompanied with SD appears to be mediated by the AKT/mTOR pathway. These results could mean that autophagy helps to relieve oxidative stress induced by SD. Our findings may be useful for elucidating the role of oxidative stress during the induction of autophagy in sleep-deprived animals or for assessing the role of autophagy in antioxidative defenses specifically in the liver. Despite previously published links between oxidative stress and autophagy, and oxidative stress and sleep deprivation fully supporting our conclusion, more experiments need to be done in the future to clarify the direct relationship among SD, oxidative stress, and autophagy.

## Data Availability

The data used to support the findings of this study are included within the article.
